# Obsessive-compulsive disorder in a child with poor tolerance to psychotropic drugs, the efficacy of psychotherapy

**DOI:** 10.1192/j.eurpsy.2025.1120

**Published:** 2025-08-26

**Authors:** M. De La Fuente, L. Cayon, M. Ruiz, P. Llano, M. Polo, R. Obeso, M. Hoyuelos, P. Hernandez, C. Sevilla, J. Romay, I. Ibarra, J. Posada, M. Martinez

**Affiliations:** 1 HUMV, Santander, Spain

## Abstract

**Introduction:**

Obsessive-compulsive disorder (OCD) in children and adolescents presents similarly to that in adults, but with some particularities. In children, the most common obsessions are usually related to fears of contamination, harm to others, or catastrophic situations. The compulsions associated are often: excessive hand washing, repetitive checking, and the need for order.

Treatment of OCD is based on both pharmacological measures and psychological therapies. At the pharmacological level, it focuses on the use of selective serotonin reuptake inhibitors (SSRIs). In terms of psychological treatment, cognitive behavioural therapy (CBT) is the psychotherapeutic intervention of first choice for the treatment of OCD, and is considered the most effective intervention, both in monotherapy and in combination with pharmacological treatment. Within CBT, the most effective is Exposure with Response Prevention (ERP).

**Objectives:**

To present the adaptation of the different therapeutic interventions so that they are effective when applied to children, through a clinical case of a 10-year-old child with OCD.

**Methods:**

Our patient first came to the emergency department due to intense anxiety, secondary to obsessive thoughts about suicide and sexual identity. Due to the incoercible anguish caused by these egodystonic thoughts and the secondary low mood, he was admitted to hospital. At the pharmacological level, sertraline was progressively administered up to a dose of 100mg/day and psychoeducation was provided. Despite the initial improvement in anxiety and hospital discharge, after a couple of weeks there was a further worsening, which persisted despite increasing the dose of sertraline. In successive outpatient consultations, psychoeducational intervention and review of confrontation techniques were carried out. The patient began with clinical symptoms typical of hypomanic turn, so SSRIs was lowered back to the initial dose, and aripiprazole 5mg was added. The importance of CBT was emphasised, teaching the parents to practice it daily with their child. In the next visits, psychotherapy work continued in the form of role-play, explaining the difference between distraction and confrontation, lowering the SSRI to 50mg and maintaining aripiprazole. Clinical stabilisation was achieved and has been maintained since.

**Results:**

We see with this case that pharmacological treatment, although necessary to obtain changes at the neuro-biological level, is not sufficient for clinical remission, and that intensive psychotherapy is the cornerstone of the intervention

**Conclusions:**

In conclusion, in the treatment of childhood OCD, the combination of pharmacological and psychological interventions is essential. Although pharmacological treatment is important, it alone does not always guarantee complete remission, highlighting the need for a comprehensive therapeutic approach to achieve sustained results over time.

**Disclosure of Interest:**

None Declared

